# FKBP52 in Neuronal Signaling and Neurodegenerative Diseases: A Microtubule Story

**DOI:** 10.3390/ijms23031738

**Published:** 2022-02-03

**Authors:** Béatrice Chambraud, Cillian Byrne, Geri Meduri, Etienne Emile Baulieu, Julien Giustiniani

**Affiliations:** 1 INSERM U1195, Université Paris-Saclay, 80 Rue du Général Leclerc, 94276 Kremlin-Bicêtre, France; beatrice.chambraud@inserm.fr; 2 Institut Professeur Baulieu, 80 Rue du Général Leclerc, 94276 Kremlin-Bicêtre, France; cillian.byrne@sorbonne-universite.fr (C.B.); geri.meduri@inserm.fr (G.M.); 3 Laboratoire des Biomolécules, LBM7203, CNRS, École Normale Supérieure, PSL University, Sorbonne Université, 75005 Paris, France

**Keywords:** FKBP52, microtubule, Tau, synuclein, neurons, Parkinson disease, Alzheimer disease

## Abstract

The FK506-binding protein 52 (FKBP52) belongs to a large family of ubiquitously expressed and highly conserved proteins (FKBPs) that share an FKBP domain and possess Peptidyl-Prolyl Isomerase (PPIase) activity. PPIase activity catalyzes the isomerization of Peptidyl-Prolyl bonds and therefore influences target protein folding and function. FKBP52 is particularly abundant in the nervous system and is partially associated with the microtubule network in different cell types suggesting its implication in microtubule function. Various studies have focused on FKBP52, highlighting its importance in several neuronal microtubule-dependent signaling pathways and its possible implication in neurodegenerative diseases such as tauopathies (i.e., Alzheimer disease) and alpha-synucleinopathies (i.e., Parkinson disease). This review summarizes our current understanding of FKBP52 actions in the microtubule environment, its implication in neuronal signaling and function, its interactions with other members of the FKBPs family and its involvement in neurodegenerative disease.

## 1. Introduction

FK506-binding proteins (FKBPs) belong to a subclass of the highly conserved immunophilin family which comprises a number of multifunctional proteins originally defined by their capacity to bind the immunomodulator FK506. FK506 binding domains harbor a Peptidyl-Prolyl *cis/trans* Isomerase (PPIase) activity that catalyzes the conversion of Peptidyl-Prolyl bonds between *cis* and *trans* conformations, resulting in a rate limiting change of protein conformation and function [1,2]. PPIase activity is known to be inhibited by immuno-suppressant molecules such as FK506 and Rapamycin via a gain of function mechanism mediating immune suppression [3,4,5]. This activity provides new perspectives to elaborate original therapeutic strategies targeting FKBPs and their ligands. First identified in human T lymphocytes, immunophilins have a wide distribution and are particularly abundant in the nervous system, suggesting unexpected and novel functions distinct from immuno-modulatory effects. Indeed, neuroprotective effects of FK506 have been reported [6,7]. In the past decade, several studies have highlighted neuronal functions of FKBPs and identified various signaling pathways in which FKBP52, a large molecular weight member of FKBPs, plays an active role. Moreover, FKBPs and in particular FKBP52, have been associated with a number of pathologies including hormone-dependent and stress related diseases [8,9], cancer [10,11] and neurodegenerative diseases [12,13,14]. In this review, we provide an overview of FKBP52 action in regulating different signaling pathways in the microtubule (MT) environment, its interchange with other members of the FKBP family, its neurological functions and involvement in neurodegenerative diseases.

## 2. FKBP52, a Member of FKBP Family Proteins

Several FKBPs have been detected in mammals and named according to their molecular mass (e.g. the 12 kDa FKBP12). The smallest FKBPs, such as FKBP12, which is the first discovered and much investigated prototypic member of the immunophilin family [5,15], are composed almost entirely of a PPIase motif in a single domain whereas the larger FKBPs are composed of functionally independent domains. All of these FKBPs show a wide distribution and are particularly abundant in the nervous system [6,16]. FKBPs are historically classed into three categories depending of their subcellular localization: Cytoplasmic, Nuclear and Endoplasmic Reticulum (ER). FKBP12 and FKBP12.6 are mostly diffuse throughout the cytoplasm [5,7] whereas larger FKBPs are localized in the ER [17,18,19,20,21,22,23], in the nucleus [24,25,26,27], in different organelles [28,29] or are associated with the cytoskeleton [30,31,32] suggesting different functions for these FKBPs (Table 1, [33,34,35,36,37,38,39,40,41,42,43,44,45,46,47,48,49,50]).

**Figure 1:**
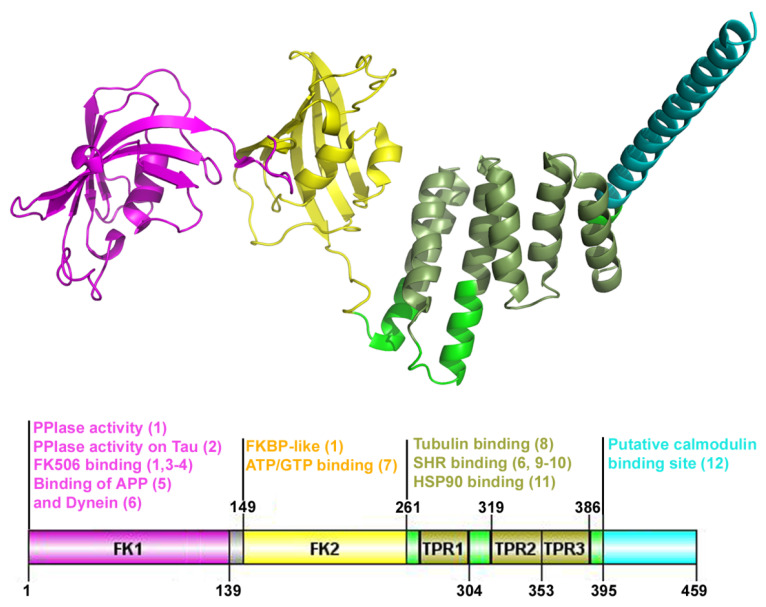
The three-dimensional structure of FKBP52 (aa1_459) showing the main structural domains. Functional domains are colored. The cartoon is generated with Pymol v0.99 and DOG 2.0. APP: amyloid precursor protein; FK: FK506 binding domain; Hsp90: heat shock protein of 90 kDa; SHR: steroid hormone receptor; TPR: tetratricopeptide repeat. References: (1) Chambraud et al, 1993; (2) Kamah et al, 2016; (3) Schreiber et al, 1991; (4) Harding et al, 1989; (5) Sanokawa-Akakura et al, 2010; (6) Silverstein et al, 1999; (7) Le Bihan et al, 1993; (8) Chambraud et al, 2007; (9) Tai et al, 1986; (10) Renoir et al, 1990; (11) Radanyi et al, 1994; (12) Massol et al, 1992.

FKBP52, one of the larger FKBPs localized both in the nucleus and the cytoplasm, is composed of four functional domains ([68]; Figure 1): two consecutive FK506-binding domains namely FK1 (residues 31–139) and FK2 (residues 149–267), a TetratricoPeptide Repeat (TPR) domain and an α-helix in its extreme C-terminus that contains a putative calmodulin binding site [69]. FK1 is the only binding site for FK506 and presents peptidyl-prolyl isomerase (PPIase) activity, while the FK2 domain, which shares 34% identity with FK1, does not bind FK506, is probably more correctly defined as an FKBP-like domain and harbors an ATP (GTP) binding site [3,70]. Another noteworthy structural aspect of the FKBP52 is the presence in the third domain of a TPR with a chaperone activity [71] which is also a binding site for the molecular heat shock protein chaperone of 90kDa (Hsp90) [72].

FKBP52 is partially associated with the MT network in different cell types suggesting an implication in MT function [30]. MTs are key cytoskeletal components that play an important role in neuronal architecture, intracellular trafficking and signaling [73,74].They are highly regulated and possess a dynamic character allowing them to continuously migrate through the cytoplasm. Originally discovered in association with steroid hormone receptors (SHR), FKBP52 is involved in endocrine signaling in a MT-dependent manner [75,76,77]. This is also the case for FKBP51, a close-related partner of FKBP52, with whom it shares 75% similarity and 60% identity [8,68,78]. In spite of their high homology, these FKBPs show different conformational dynamics [79] and both immunophilins usually compete for binding to target proteins, have competing functional properties and elicit different biological effects [57,80]. However, some exceptions have been described showing a redundancy of some effects of these FKBPs [58,64]. 

## 3. FKBP52 in Microtubule Dynamics 

In accordance with previous reports about the localization of FKBP52 in association with the microtubular network in mouse neuronal cells [30], our group has shown that FKBP52 is able to interact with the neuronal tubulin preventing in vitro MT formation [31]. The inhibition of MT polymerization involves the C-terminal region of FKBP52 and is independent of its PPIase activity. Consistent with these observations, FKBP12 has no effect on tubulin polymerization in vitro. Likewise, depletion of FKBP52 stimulates neurite extension in an undifferentiated PC12 rat pheochromocytoma cell line suggesting that FKBP52 could contribute to neurotrophic effects via its regulation of MT dynamics [31]. Conversely, certain results showed that neurite outgrowth in vitro in embryonic hippocampal cells was favored by FKBP52 overexpression [81]. This discrepancy might be explained by a possible cell type-dependent role of FKBP52. FKBP52 was also found to associate with canonical transient receptor potential channels TRPC1 and to control chemotropic guidance of neuronal growth cones, which implicates the rearrangement of the cytoskeleton, via the regulation of TRPC1 channel opening [82]. As TRPC1 was shown to inhibit Ca^2 +^ influx and as calcium plays an important role in MT depolymerization [83], FKBP52 might indirectly modulate MT dynamics locally in the growth cones. In the same vein, FKBP52 was found to interact with TRPC3 [84] and TRPV5 [85] in different epithelial cells, and to inhibit Ca^2+^ influx but their neuronal involvement still remains to be analyzed. Moreover, we have found that FKBP52 interacts with Tau (Tubulin associated unit), a neuronal protein implicated in tubulin polymerization, and have demonstrated an antagonist effect of FKBP52 on Tau promoted tubulin assembly [12]. In contrast to the effect of FKBP52, it has been shown that FKBP51 promotes MT stabilization via its interaction with Tau and Hsp90 [63]. Altogether, these observations suggest an antagonistic action of these two FKBPs on the regulation of Tau-dependent MT dynamics in neurons. FKBP25 is also able to promote tubulin polymerization and stabilize the MT network in various cell lines but its involvement in neurons still remains to be investigated [32]. Moreover, another large FKBP, called FKBP133, has been localized in the neuronal microtubular network and has been suggested to play a role in membrane trafficking and axonal outgrowth [27,67]. Additional studies need to be done to evaluate the involvement of FKBP25 and FKBP133, and also of the other FKBPs previously listed in Table 1, in MT formation and particularly in Tau-induced tubulin polymerization. All these findings taken together indicate the implication of some FKBPs including FKBP52 in MT dynamics and their place in the large family of cytoskeleton-modulating proteins.

## 4. FKBP52 in Microtubule-Dependent Trafficking 

In addition to its implication in MT dynamics, FKBP52 has been shown to play important roles in protein trafficking along the MT network, regulating various physiological processes to maintain brain homeostasis. For example, it has been observed in different cell lines including neurons that FKBP52, in close interaction with Hsp90 and the dynein/dynactin complex, is implicated in the retrograde transport and translocation of different steroid receptors such as the glucocorticoid receptor (GR) [57,77,80,86,87]. Glucocorticoids act as peripheral effectors of the hypothalamic-pituitary-adrenal (HPA) axis and interact with the GR modulating response to stress and inflammation, synaptic physiology and circuitry, and also behavior [88]. While the TPR domain of FKBP52 binds to the C-terminal EEVD peptide motif in Hsp90 [89,90], the PPIase domain interacts with the dynein complex [86]. This complex binds with high affinity the hormone bound activated GR inducing its nuclear translocation and mediating its transcriptional response [91]. In contrast, FKBP51 does not interact with dynein and, through competition with FKBP52, is able to disrupt the GR-dynein association thereby impairing nuclear translocation [80]. The latter study describes an antagonistic action of FKBP52 and FKBP51 in the regulation of GR signaling as observed in neuronal MTs dynamics. It has also been shown that FKBP12 does not compete with FKBP52 for dynein binding suggesting that FKBP12 is not involved in GR translocation [86]. Similarly to FKBP52, FKBP-L is an additional player in the Hsp90/Dynein/GR complex leading to GR nuclear translocation and transcriptional activity [50]. 

It was also shown that both FKBP52 and FKBP51 modulate the nuclear translocation of the p50.RelA/p65 complex and affect the transcriptional activity of Nuclear Factor K-light chain enhancer of activated lymphocyte B cells (Nf-kappaB) which plays a key role in inflammation and immune function [59,92,93,94]. Nf-kappaB is translocated to the nucleus by a MT-dependent mechanism in hippocampal neurons analogously to the nuclear shuttling occurring during steroid hormone signaling to regulate expression of several genes involved in neuroplasticity and cell survival [95]. In T and B lymphocytes, FKBP52 inhibits IRF-4 (interferon regulatory factor 4) transactivation via its PPIase activity and possibly acts as a chaperone to escort IRF-4 into the nucleus by the same MT-dependent transport. However, the latter action still remains to be investigated [60]. In neurons, IRF4 is a neuroprotective factor from ischaemia/reperfusion-induced degeneration and apoptosis both in vivo and in vitro [96]. It has also been shown in rat dorsal root ganglion neurons that FKBP52 interacts with Atox1 (Antioxidant Protein 1), a copper-binding metallochaperone,, and is involved in copper transport [97]. This transport might also be mediated through the same FKBP/Dynein complex along a MT lattice [98] and possibly plays a role in inflammation processes [99]. These observations indicate that large FKBPs including FKBP52 are involved in neuroplasticity, inflammation and cell survival, possibly through regulation of MT protein trafficking as shown for GR and Nf-KappaB signaling.

The same FKBP52/Dynein complex is involved in the retrograde transport of both the tumor suppressor p53 [100,101] and the human telomerase reverse transcriptase hTERT [58] along the MT network promoting their nuclear translocation and transcriptional activity; however, their possible involvement in neuronal functions still remains to be elucidated. Recently, we have shown that FKBP52 is located in the endo-lysosomal system of different neuronal cells and that FKBP52 deficiency impacts the lysosomal positioning during a proteotoxic stress [29,61]. Knowing that lysosomal movement toward the nucleus is MT-dependent and mediated by dynein motors [102], we hypothesized that the role of FKBP52 in protein trafficking might be also extended to the retrograde lysosomal transport [61]. In the same way, FKBP133 depletion results in altered endosomal transport towards the lysosomes suggesting an implication of FKBPs in membrane trafficking [67]. FKBPs and particularly FKBP52 are thus involved in protein and membrane trafficking along the MT network which regulates MT-dependent signaling dynamics in the cytoplasm. 

## 5. FKBP52 in Microtubule-Associated Protein Aggregation and Clearance

MT stability and function are highly regulated by different MT-associated proteins including some neuronal aggregation-prone proteins such as Tau and alpha-synuclein (α-syn). Both proteins have been shown to polymerize MTs in vitro [103,104,105,106] and to be involved in MT-dependent trafficking [107,108]. Both proteins can also aggregate and generate toxic intracellular deposits that induce MT alterations and neuronal death [109,110]. Whereas protein aggregation is closely associated with neuronal toxicity, it has been suggested that protein aggregates could also represent a neuronal protective response during stress depending on the nature, the size and also the neuronal load levels of these aggregates [111,112,113].

In addition to its regulation of Tau-induced tubulin polymerization, we have recently demonstrated that FKBP52 is also able to modulate Tau aggregation in vitro [54,55]. These studies show that FKBP52 has no effects on full-length Tau aggregation (HT40 isoform), whereas we demonstrate a distinct FKBP52 activity upon truncated and mutated Tau forms independent of its PPIase activity [56]. Similarly, it has been shown that FKBP52 is also able to accelerate α-syn aggregation [13,51,52]. Other FKBPs can also affect the oligomerization and aggregation of Tau and α-syn but the physiological role of this particular action still remains elusive [33,62,114] (see Table 1). 

FKBP52 and FKBP51 are known to interact with Hsp90 acting as co-chaperones to maintain protein quality control during stress [9]. It has been reported that Hsp90 regulates Tau function [115,116] and synergizes with FKBP51 to block Tau degradation resulting in Tau oligomerization under pathological conditions [63,114]. Whereas some chaperones/co-chaperones stimulate Tau aggregation in vitro, others are thought to inhibit or reverse this process suggesting that this chaperone machinery might regulate the levels of Tau aggregation [117,118,119]. Different chaperones have also been found to modulate α-syn aggregation without evidence of synergy with FKBPs in this aggregation process [120,121]. 

Both Tau and α-syn aggregates are known to be degraded through the autophagy-lysosomal pathway (ALP) [122,123]. ALP is a MT-dependent degradation pathway involved in the clearance of abnormal protein aggregates in order to maintain protein homeostasis and neuronal health [124]. Indeed, MTs are involved in different steps of autophagy including motility of autophagosomes and lysosomes using MT-interacting molecular motors such as dynein and kinesins [125,126]. One possible explanation of FKBP implication in the control of protein aggregation might be found in their involvement in neutralizing soluble and toxic oligomeric forms of Tau, and/or α-syn, inducing their degradation and clearance under stress conditions. Our recent observations showed a localization of FKBP52 in the lysosomes of healthy human brain neurons [29,61]. In the latter study, we showed that FKBP52 modulates ALP function during Tau-induced proteotoxic stress by modulating perinuclear lysosomal positioning and clustering, an important lysosomal process involved in protein degradation during stress [61]. Interestingly, recent studies have also reported the involvement of other FKBPs in ALP function [65,66]. These results might suggest a synergistic role of FKBP52 in the MT environment (1) through regulation of MT-dependent aggregation of proteins such as Tau and α-syn in association with other chaperones and/or FKBPs and (2) through contributing to the efficient degradation and clearance of these protein aggregates via the ALP during stress. 

## 6. Involvement of FKBP52 in Neurodegenerative Diseases

Abnormalities in neuronal MT functions such as network stability and defective transport along the axon lead to brain diseases [127]. Indeed, alterations and aggregation of Tau and α-syn, which both impact MT assembly and signaling, are critical for the development of major neurodegenerative diseases called tauopathies including Alzheimer disease (AD) and synucleinopathies such as Parkinson disease (PD) respectively [128,129,130]. In these pathologies, the loss of MT function is accompanied by a gain of toxicity of Tau or α-syn both leading to neuronal death [131,132,133,134,135]. The pathogenesis of AD is also believed to be linked to an abnormal production of the β-amyloid peptide (Aβ) produced through the proteolytic processing of a transmembrane protein called amyloid precursor protein (APP) and which accumulates extracellularly, forming amyloid plaques [136,137]. It has been shown that Aβ peptides cause rapid MT loss in a Tau-dependent manner in cultured neurons linking Aβ and Tau to the detrimental neurodegeneration observed in AD [138]. Given the important role of FKBP52 and other FKBPs in the MT environment, a different modulation of their expression and/or a deviance of their function under pathological conditions are thought to contribute to the alterations of MT-dependent signaling dynamics thus impacting neuronal function and health. FKBP52 protein expression is strongly decreased in the frontal cortex of AD and FTLD-Tau brains (Frontotemporal Lobar Degeneration linked to a point-mutation of the Tau gene (i.e Tau-P301L)) [139] whereas no indication has been provided to date concerning the expression of FKBP52 in affected brain regions of PD patients. It has also been reported that the expression of other FKBPs is altered in these pathologies. For example and in contrast to FKBP52, FKBP51 is increased in different brain regions of patients with AD including the frontal and temporal cortex [114]. The latest observation is consistent with the recent study showing the ability of FKBP51 to modulate the expression of FKBP52 in the mouse brain and speculating for an inverse correlation between FKBP51 and FKBP52 expression levels in human brain [140]. FKBP12 immunoreactivity is also increased in the hippocampus and the frontal cortex of patients with AD and PD [16] whereas its expression is decreased in angular cortices of AD brains [53]. 

### 6.1. FKBP52 and Microtubule Dynamics

The P301L mutation of the Tau gene, which has been observed in patients with FTLD-Tau [141], results in a reduced ability of Tau to promote MT assembly. This mutation is linked to abnormal axonal outgrowth and branching in defective spinal primary motoneurons in the hTau-P301L zebrafish model [142,143]. Our team has shown that the FKBP52 interaction with Tau (both wild-type and TauP301L) has a considerable impact on the progression of the early manifestations of the tauopathy in vivo, because in the model of hTau-P301L zebrafish mutant used for the study, the early axonal growth defects are rescued by FKBP52 knock down [54]. Given that FKBP52 is able to prevent Tau-induced tubulin polymerization and MT formation [12,31], reducing its interaction with wild-type Tau probably improves the defective MT dynamics and stability engendered by the mutated Tau. However, reducing the levels of FKBP52 does not decrease neuronal toxicity in the spinal cord of hTau-P301L zebrafish larvae, but rather tends to increase neuronal cell death in this pathological context. These results underline the importance of maintaining normal FKBP52 expression levels on neuronal survival and suggest a double-edged sword function of FKBP52 in this neurodegenerative model [54]. 

### 6.2. FKBP52 in Protein Aggregation and Clearance

FKBP52 decrease in AD brains is correlated with the accumulation and aggregation of pathological Tau [29]. In our previous studies, we proposed that FKBP52 might play a role in Tau degradation [12,139], and we showed that FKBP52 is present in the endo-lysosomal system of human brain neurons [61]. As previously discussed, FKBP52 is able to promote in vitro oligomerization of different truncated or mutated Tau species [54,55] and to accelerate α-syn aggregation [13,51,52]. In the case of Tau proteins, it has been shown that amyloidogenic Tau fragments, expressed in a mouse neuroblastoma cell line, are targeted to the lysosome surface where they aggregate to be eventually degraded through the ALP [122]. Thus, we propose that FKBP52 might participate to the aggregation of Tau at the surface of the lysosome, leading to Tau degradation through the ALP. This particular FKBP52 action might possibly expand to other aggregation-prone proteins such as α-syn. If we consider that FKBP52 modulates Tau aggregation synergistically with other FKBPs/chaperones in the lysosomal environment in order to degrade these aggregates, we might also suppose that its decreased expression in AD brain neurons could disturb this highly regulated process. Tau oligomers/small aggregates would escape in the cytoplasm during their lysosomal degradation thus progressively propagating abnormal and uncontrolled Tau aggregation in a prion-like manner as described [144,145,146]. Moreover, the recent observations showing an involvement of FKBPs in ALP function highlight a possible synergistic role of these FKBPs in Tau aggregation and clearance [61,65]. Indeed, we recently demonstrated that FKBP52 deficiency impairs autophagy facilitating Tau accumulation during Tau-induced proteotoxic stress in a human neuronal cell line and in TauP301S dorsal root ganglion neurons, suggesting that the decrease of FKBP52 detected in AD neurons might be relevant to the progression of the tauopathy [61]. Due to its involvement in several neuronal MT-dependent signaling pathways, FKBP52 overexpression can also have severe side effects as described by recent studies on different mouse models [147,148] pointing again to the importance of preserving normal FKBP52 expression levels in the brain for neuronal health. Nevertheless, FKBP52 is a highly expressed protein in brain neurons compared to glial cells [29] and its neuronal overexpression and specific contribution to neuronal homeostasis still remain to be analyzed. On the other hand, FKBP52 also binds APP and its overexpression decreases the toxicity associated with the transgenic expression of Aβ peptides in Drosophila which is possibly due to the involvement of FKBP52 in Aβ turnover [149]. This inverse effect of FKBP52 overexpression on Aβ and Tau toxicity might be due to the level or the timescale of FKBP52 expression in these models. 

### 6.3. FKBP52 in Stress and Inflammation

As previously described, FKBP52 is implicated in the retrograde transport and translocation of the hormone bound GR [57]. Abnormal FKBP52 decrease in AD and FTLD-Tau brain might also induce an imbalance in GR signaling and modulate glucocorticoid levels thus impacting on responses to stress and inflammation. Neuronal exposure to high levels of glucocorticoids is known to be a major risk factor of AD and various studies have shown that glucocorticoids cause abnormal Tau and Aβ accumulation leading to MT and synaptic dysfunction [150,151,152]. Upon ligand binding, GR is translocated to the nucleus with the help of FKBP52 regulating gene expression which may result in the inhibition of pro-inflammatory genes in glial cells [153]. Nuclear translocated GR is able to form heterocomplexes with Nf-kappaB, a transcription factor involved in the expression of various pro-inflammatory genes, to inhibit the biological response of Nf-kappaB through transrepression [59]. Hence, deregulated FKBP52 expression in glial cells might be involved in the disruption of the anti- and pro-inflammatory signaling balance resulting in chronic neuroinflammation and neuronal damage as observed in pathologically vulnerable regions of AD brain [154,155,156]. Another possible effect is FKBP52 interaction with Atox1 and it has been shown that FKBP52 overexpression increases rapid copper efflux in copper-treated cells suggesting that FKBP52 might protect neurons against copper toxicity [97]. Copper is a metal ion implicated in the pathogenesis of AD and PD [157,158] and is an essential micronutrient that plays a fundamental role in innate and adaptive inflammation again suggesting a possible FKBP52 involvement in this process [99]. 

## 7. Conclusions

FKBP52 is a ubiquitously expressed and highly conserved protein that plays important roles in the MT environment. Various studies highlight its importance in several MT-dependent signaling pathways such as protein transport, transcriptional regulation, protein aggregation and clearance, and suggest that FKBP52 plays a key role in neurite outgrowth, neuronal differentiation, inflammation, endocrine stress responses and cellular homeostasis. Given its involvement in MT signaling dynamics and protein aggregation, FKBP52 has been implicated in several neurodegenerative diseases such as AD and PD and represents a promising theranostic target in these pathologies. Our current knowledge tends to show that variations of brain FKBP52 expression contribute to the progression of Tau pathogenesis and points to the importance of preserving physiological FKBP52 expression levels for neuronal survival. FKBP52 also jointly acts with other members of the FKBP family as shown by the antagonistic action of FKBP52/FKBP51 in regulating MT dynamics and protein trafficking in neurons or by the cooperative effects of FKBPs in controlling Tau aggregation. Efforts to improve our understanding of the role and interplay of each FKBP in MT function and of their involvement in aggregation-prone protein accumulation and clearance would enhance our knowledge on their impact in the neurodegenerative processes. Deciphering the implications of FKBPs in neuronal homeostasis and pathology could assign them the role of therapeutic targets for the treatment of the neurodegenerative diseases linked to pathologic protein aggregation.

## Figures and Tables

**Table 1:** List of human FKBPs: cellular location, function and central nervous system (CNS) disease association.

Gene	FKBPs	FK506 Binding/PPIase Activity	Cellular Location	Cellular Function	CNS Expression	CNS Disease Association
*FKBP1a*	FKBP12	Yes/Yes	Cytoplasm	- Regulates Tau aggregation [33] - Regulates APP processing [34] - Increases α-Syn aggregation [13,51,52] - Regulates ryanodine receptors (RyRs) [35]	-Detected in all brain regions and spinal cord -Expressed in Neurons and dystrophic neurites [53]	- Tauopathies (AD) - α-Synucleinopathies (PD)
*FKBP1b*	FKBP12.6	Yes/Yes	Cytoplasm	- Regulates ryanodine receptors (RyRs) [35]	Detected in all brain regions and spinal cord	AD [35]
*FKBP2*	FKBP13	Yes/Yes	Endoplasmic reticulum	-ER Chaperone [20]	Detected in all brain regions and spinal cord	Unknown
*FKBP* *3*	FKBP25	Yes/Yes	Nucleus and cytoplasm	- Regulates MT dynamics and is involved in Nucleus-Cytoplasm shuttling [32] - Regulates p53 signaling [36]	- Detected in all brain regions and spinal cord -Expressed in neurons	Unknown
*FKBP* *4*	FKBP52	Yes/Yes	Cytoplasm, nucleus, MT network, endo-lysosomal system	- Regulates Tau aggregation [54,55,56] - Increases α-Syn aggregation [13,52] - Regulates MT dynamics [12,31] - Regulates MT-dependent trafficking (i.e., SHR, Nf-kappaB, IRF4, hTERT) [57,58,59,60] - Involved in ALP function [61]	- Detected in all brain regions and spinal cord - Highly expressed in neurons	- Tauopathies (AD) - α-Synucleinopathies (PD)
*FKBP* *5*	FKBP51	Yes/Yes	Cytoplasm, nucleus, MT network, mitochondria	- Regulates Tau aggregation [62] - Regulates MT dynamics [63] - Regulates MT-dependent trafficking (i.e., SHR, Nf-kappaB, hTERT) [57,59,64] - Involved in ALP function [65]	- Detected in all brain regions and spinal cord - Highly expressed in neurons	- Tauopathies (AD) - α-Synucleinopathies (PD) - Huntington disease [37] - Stress-related and psychiatric diseases [8]
*FKBP* *6*	FKBP36	No/Yes	Cytoplasm and nucleus	- Regulates GAPDH signalling [46] - Involved in spermatogenesis [38]	Undefined	- Williams-Beuren syndrome [39]
*FKBP* *7*	FKBP23	Undefined/Yes	Endoplasmic reticulum	- Calcium binding ability [23] - Regulates interaction with BiP [40]	- Detected in all brain regions and spinal cord	Unknown
*FKBP8*	FKBP38	No/Yes	Mitochondria	- Involved in mitophagy [66] - Inhibits apoptosis [47] - Endogenous inhibitor of mTOR [41]	- Detected in all brain regions and spinal cord -Expressed in neurons	Unknown
*FKBP9*	FKBP60	Yes/Yes	Endoplasmic reticulum	- Possible role in prion propagation or clearance [42]	Detected in all brain regions and spinal cord	Unknown
*FKBP* *10*	FKBP65	Yes/Yes	Endoplasmic reticulum	- Possible neuroprotective effect linked to regulation of protein folding [43] - Participates in type I procollagen folding [44]	Detected in all brain regions and spinal cord	Unknown
*FKBP11*	FKBP19	Yes/Yes	Cytoplasm, Endoplasmic reticulum	-Protein folding and secretion [21]	Detected in all brain regions and spinal cord	Unknown
*FKBP14*	FKBP22	Yes/Yes	Cytoplasm, Endoplasmic reticulum	- Involved in protein folding, trafficking and in collagen synthesis [22] - Regulates Presenilin and Notch signalling [45]	Detected in all brain regions and spinal cord	Unknown
*FKBP15*	FKBP133	No/No	Cytoplasm, nucleus and endosome	- Possible role in cytoskeletal organization of neuronal growth cones [67] - Involved in early endosomes transport [67]	Detected in all brain regions and spinal cord	Unknown
*AIP*	FKBP37	No/No	Cytoplasm	- Interaction with Hsp90 [49] -Regulates aromatic hydrocarbon receptor signalling [49]	Detected in all brain regions and spinal cord	- Pituitary adenoma
*FKBPL*	FKBP-L	No/No	Cytoplasm	- Regulates MT-dependent trafficking of GR [50]	Detected in all brain regions and spinal cord	Unknown

## Data Availability

The data that support the findings are in the referenced studies.

## Abbreviations

AD	Alzheimer disease
ALP	autophagy lysosomal pathway
APP	amyloid precursor protein
Atox1	antioxidant protein 1
Aβ	amyloid beta
ER	endoplasmic reticulum
FK1	FK506 binding domain 1
FKBPs	FK506-binding proteins
FTLD-Tau	frontotemporal lobar degeneration linked to a point-mutation of Tau gene
GR	glucocorticoid receptor
Hsp90	heat shock protein of 90 kDa
hTERT	human telomerase reverse transcriptase
IRF4	interferon regulating factor
MT	microtubule
Nf-kappaB	nuclear factor K-light chain enhancer of activated lymphocyte B cells
PD	Parkinson disease
PPIase	peptidyl-prolyl *cis trans* isomerase
SHR	steroid hormone receptor
Tau	tubulin associated unit
TPR	tetratricopeptide repeat
α-syn	alpha-synuclein
